# Social cohesion emerging from a community-based physical activity program: A temporal network analysis

**DOI:** 10.1017/nws.2020.31

**Published:** 2020-08-06

**Authors:** Ana María Jaramillo, Felipe Montes, Olga Lucía Sarmiento, Ana Paola Ríos, Lisa G. Rosas, Ruth Hunter, Ana Lucía Rodríguez, Abby C. King

**Affiliations:** 1 Department of Industrial Engineering, Social and Health Complexity Center, Universidad de Los Andes, Bogotá, Colombia; 2 Department of Computer Science, University of Exeter, Exeter, UK; 3 School of Medicine, Universidad de Los Andes, Bogotá, Colombia; 4 Grupo de Cuidado Cardiorrespiratorio, Universidad Manuela Beltrán, Bogotá; 5 Palo Alto Medical Foundation Research Institute, Palo Alto, CA, USA and Stanford University, Palo Alto, CA, USA; 6 United Kingdom Clinical Research Collaboration Centre of Excellence for Public Health/Centre for Public Health, Queen’s University Belfast, Belfast, UK; 7 Department of Psychology, Health Disparities and Cultural Identities Research Lab, Florida International University, Miami, FL, USA; 8 Stanford Prevention Research Center, Department of Medicine, School of Medicine at Stanford University, 1070 Arrastradero Road, Suite 100, Palo Alto, CA, USA and Department of Health Research and Policy, School of Medicine at Stanford University, Palo Alto, CA, USA

**Keywords:** social network analysis, temporal networks, community-based interventions, socially transmitted conditions, physical activity

## Abstract

Community-based physical activity programs, such as the Recreovía, are effective in promoting healthy behaviors in Latin America. To understand Recreovías’ challenges and scalability, we characterized its social network longitudinally while studying its participants’ social cohesion and interactions. First, we constructed the Main network of the program’s Facebook profile in 2013 to determine the main stakeholders and communities of participants. Second, we studied the Temporal network growth of the Facebook profiles of three Recreovía locations from 2008 to 2016. We implemented a Time Windows in Networks algorithm to determine observation periods and a scaling model of cities’ growth to measure social cohesion over time. Our results show physical activity instructors as the main stakeholders (20.84% nodes of the network). As emerging cohesion, we found: (1) incremental growth of Facebook users (43–272 nodes), friendships (55–2565 edges), clustering coefficient (0.19–0.21), and density (0.04–0.07); (2) no preferential attachment behavior; and (3) a social cohesion super-linear growth with 1.73 new friendships per joined user. Our results underscore the physical activity instructors’ influence and the emergent cohesion in innovation periods as a co-benefit of the program. This analysis associates the social and healthy behavior dimensions of a program occurring in natural environments under a systemic approach.

## 1. Introduction

In low- and middle-income countries, non-communicable diseases cause 48% of annual deaths, which are mainly attributed to health-related behaviors (Allen et al., 2017; Riley et al., 2017). The World Health Organization promotes physical activity as an effective behavior to prevent non-communicable diseases (Basu & Dutta, 2008), recently reframed as socially transmitted conditions (Allen & Feigl, 2017).

It is crucial to take into account that the environment can profoundly affect health outcomes and behaviors. An interplay of individuals’ attributes, behaviors, and the structure of the social interactions in the places where they occur frame those environments (El-Sayed et al., 2012). Developing programs that employ social structures already inherent in our communities may provide a cost-efficient way of encouraging individuals to engage in regular physical activity through social support and cohesion (Hoehner et al., 2008; Shelton et al., 2019).

Socially activated interventions, such as community-based programs of physical activity, incentivize healthy habits using inherent social structures like social networks. These programs provide a focal point for interconnecting social structures, for example, families, neighborhoods, and local culture (Hunter et al., 2018) and local environments and policies, for example, parks, public policies (McLeroy et al., 2003; Samuels et al., 2010). An example of a community-based program is the *Recreovía* (in English: recreation on the streets) program of Bogotá, Colombia. The Recreovía program provides a unique opportunity for promoting physical activity in public spaces around the city by providing free physical activity classes in public spaces during weekdays and Sundays (Díaz del Castillo et al., 2017). This type of program has scaled globally and currently occurs in 45% of the Latin American countries in which they have been introduced (Diaz del Castillo et al., 2017). The evaluation of the *Recreovía* program has applied the socioecological framework and social cognitive theory (Barradas et al., 2019; Torres et al., 2017). A policy analysis has shown that the influence of the community, the governmental support, and the cultural context of these community-based programs can contribute to promoting physical activity in Colombia and Brazil (Paez et al., 2014).

The *Recreovía* program has been associated with health and social benefits, including the promotion of physical activity, mainly in women (Sarmiento et al., 2017), as well as increased quality of life related to health and life satisfaction (Barradas et al., 2019; Sarmiento et al., 2017). In terms of weekly minutes of leisure-time-moderate to vigorous physical activity, women who are regular attendees are 2.4 times more physically active than those who do not participate in the program (Torres et al., 2017). For two out of five attendees, the *Recreovía* was reported as the only place to engage in regular physical activity (Torres et al., 2017). The *Recreovía* has an approximate cost of 0.8 USD to the government per attendee per 45-minute class per year and offers notable benefits to public health (Torres et al., 2017). In light of its potential as a community-based physical activity intervention, an evaluation of the structure of the *Recreovía’s* social network can provide useful insights into the challenges that can affect its sustainability, scalability, and the social benefits of such physical activity programs (Díaz del Castillo et al., 2017).

Participation in the Recreovía could promote an increase in social capital in the community, thanks to citizen mobilization and the inclusion of attendees (Pratt et al., 2012; Sarmiento et al., 2017). For this current study, we refer to social capital as the essential resource of friendship among the participants of the program (Coleman, 1988). Previous research has shown that increased social capital raises commitment within communities along with their ability to create collective actions and resources due to the existence of cohesive relationships (Villalonga-Olives & Kawachi, 2015). Also, some studies have shown how this form of social capital allows the flow of useful information sharing, an increase in personal relationships, and the capacity to organize groups from the network (Ellison et al., 2007; Zarama et al., 2011).

Studying the social networks to more clearly understand the social structures of community-based programs like the Recreovía can better guide the implementation and sustainability of such community-based interventions (Hunter et al., 2018). For example, a network science study analyzed the network structure of interconnected organizations that form a parallel program that promotes physical activity—the *Ciclovía* of Bogotá (Meisel et al., 2014). This program was found to be an emerged, self-organized system of institutions that work together to establish a free, multicultural, community-based physical activity program (Meisel et al., 2014). However, there are no studies to date about the interaction of their stakeholders, including attendees, physical activity instructors, coordinators, and external agents involved in the program.

The current study aims, for the first time, to: (1) characterize the social networks of stakeholders in a community-wide physical activity program in Latin America using Facebook data; (2) using social network analyses, to evaluate the potential ability of the physical activity program to generate social capital through the cohesion and social interaction of program participants. For that purpose, we first describe the existing social network around the Facebook profiles of the Recreovía program to evaluate if this network was highly cohesive, with multiple communities of which physical activity instructors are the central nodes. Furthermore, we assess the social cohesion among *Recreovía* participants through the change in network structure and characteristics over time. This provides us evidence to evaluate if through time new friendships among program attendees would emerge as evidence of cohesion and social capital. Finally, we analyze our results with implications for community-based physical activity interventions using social structures.

This analysis could serve as an example for other community programs of the potential of social network analysis to provide a greater understanding and monitoring of the social dimension of physical activity programs. The findings can also help to clarify the characteristics of the key stakeholders and agents of change in the social network, which will improve the information given to policymakers and practitioners for future programs.

## 2. Methods

### 2.1 The Recreovía program

The *Recreovía* program of Bogotá started in 1995 as a program providing free recreational activities, especially for socioeconomically vulnerable residents (Rios et al., 2017). The *Recreovía* program offers free physical activity classes led by trained physical activity instructors on weekdays, Sundays, and holidays. The physical activity classes include aerobics with a cultural dancing component (e.g. salsa, rumba, merengue, reggaeton, and folk dance), strength training and stretching for adults, and other sessions for children. The program is currently being implemented in 67 settings throughout Bogota reaching 19 of 20 administrative localities (Díaz del Castillo et al., 2017).

### 2.2 Data collection

To collect social network data, we used four different Recreovía Facebook accounts. The accounts’ information was provided by the local authority for sports and recreation—District Institute of Recreation and Sports (acronym in Spanish: IDRD).

The first account corresponded to the leading Facebook group of the Recreovía. The program had its first Facebook group account starting in 2008, and we collected its friendship network in 2013. We used the Facebook application Netvizz to gather public and available data about group members and their interactions in the Facebook group (Rieder, 2013). We collected these data in 2013 when the Facebook data policies permitted us to record the sex of nodes as the only demographic variable. From this point forward, these network data will be referred to as the Main network.

The other three Recreovía accounts corresponded to the Facebook profiles of three specific Recreovía locations in the city. We selected these locations because they were the intervention group from an intervention project conducted during the 2013–2015 period called “Al ritmo de las comunidades” (Sarmiento et al., 2017; Torres et al., 2017). In 2013, the attendees of these locations were encouraged to add the Facebook account of the *Recreovía* location where they were attending. The locations were as follows:

1. Recreovía Valles de Cafam: inaugurated in October of 2011, suspended in 2012, and reinaugurated in October of 2013. It has a Facebook account from 2011.
2. Recreovía Santa Isabel: inaugurated in October of 2010, suspended in 2011, and reinaugurated in February of 2014. It has a Facebook account from 2010.
3. Recreovía Meissen: inaugurated in September of 2013 and suspended in 2015. It has a Facebook account from 2014.

We collected the temporal Facebook friendship networks from each of the three program location accounts in October 2016 by using the Google Chrome extension Lost Circles. This extension built the social network of a Facebook profile where nodes represented friends of a Facebook account, and ties represented common friendships between Facebook friends (Lost Circles, n.d.). To analyze the structure of the friendship networks, we defined the nodes as Facebook friends of the Recreovía account and we collected, when available, individual characteristics such as name and job in their Facebook profiles. The edges (connections among nodes) were defined as mutual Facebook friendships, and we collected the starting date of each friendship. Then, we aggregated the three networks, obtaining a unique temporal network from this point forward referred to as the *Temporal network.*

Finally, in 2016 we surveyed, in person, 50 attendees of the *Recreovía* program in 2 locations—Valles de Cafam (*n* = 44, 88%) and Santa Isabel (*n* = 6, 12%)—to assess the physical environment and the offline spaces where attendees interact. The *Meissen* location was excluded from this data collection because the program stopped operating in this park in 2015 due to budget cuts.

The procedure for the interviews was conducted on two consecutive Sundays for each park. Weekend days were selected based on the recommendations of the physical activity instructors, as the days with more attendees. Interviews were conducted during the breaks either before or after each class. Physical activity instructors announced to the attendees that interviews would take place during the break time without interfering with the regular schedule. Eligible participants were adults aged 18 years or older living in Bogota and without cognitive deficit or physical disability. Participants who provided verbal consent answered the questionnaire, which lasted about 5 minutes.

We asked attendees about offline spaces outside of the classes that they shared with their program friends, and their Facebook use to obtain information about the Recreovía. The survey included the following questions: How do you interact with other attendees of the *Recreovía*? How much do you use Facebook to interact with other attendees of the *Recreovía?* Where do you interact with other attendees of the *Recreovía?* Are you a “friend” in Facebook of this *Recreovía* location?

The protocol of the study was approved by the Universidad de Los Andes ethics committee in their Acts No. 208 of 2013 and 751 of 2017.

### 2.3 Assessing cohesion of the human system of the program with network measurements

We recorded three variables to measure the cohesive properties of the network, as follows: (1) the density, calculated as the number of observed friendships over the number of all possible friendships in the network (Barnes, 1968); (2) the diameter, calculated as the length of the maximum shortest distance among a pair of individuals (Wasserman & Faust, 1995); and (3) the average clustering coefficient, calculated as the average probability that two friends of an individual are also friends forming a triad (Watts & Strogatz, 1998).

We measured cohesion using network structure parameters reflecting the network connectivity patterns (Moody and White, 2003). A cohesive network has high density, small diameter, and high average clustering coefficient as evidence of a high volume of relationships, a short path between all nodes in the network, and stronger groups reflected in more probable triads’ formation.

### 2.4 Identifying influential stakeholders and communities in the human system of the program

For the Main network, we analyzed its topological and structural properties to understand how to foster human cooperation through the emerging social interactions of the program participants. To identify communities, principal communities of stakeholders, and influential individuals, we detected the community structure of the network using the Louvain algorithm (Blondel et al., 2008). We identified communities only for the Main network due to its cross-sectional and static data, and we selected this algorithm to detect communities as node groups with dense internal connections and sparse external connections. A quality function over those group divisions is modularity (Newman and Girvan, 2004), and the Louvain algorithm optimizes it (Blondel et al., 2008).

Subsequently, with the coordinator of the *Recreovía*, we assigned a label for each community by identifying their members. For the largest community, we conducted a sub-analysis to identify the main stakeholders. These nodes can be labeled as community hubs: the most connected nodes in each community (Montes et al., 2020). Here, we identified those community hubs as nodes with more than 300 friends in the largest community.

To identify influential stakeholders for targeting interventions in the *Recreovía* program, we used well-known seed node strategies based on centrality metrics (Freeman, 1978). We selected the top-ranked nodes of each metric, and we measured the overlap among metrics and identified the role of those nodes. We recorded the degree centrality as the number of connections (Freeman, 1978), betweenness centrality as the number of times being in the shortest path between two nodes in the network (Freeman, 1978), closeness centrality as the geodesic distance to all nodes of the network (Freeman, 1978), k-core centrality as the deepest core to be removed in the network (Seidman, 1989), and page rank as the probability of appearing in a random walk on the network (Page, 1998).

### 2.5 Assessing the emergence of social cohesion in the program over time

For the Temporal network, we evaluated the emergence of cohesion among stakeholders over time. We analyzed the Temporal network detecting the meaningful length of time windows; then, we assessed the cohesion scaling by tracking the network growth through the time windows.

#### 2.5.1 Identifying time windows for the dynamical human system of the program

We determined the optimal time window length to analyze the network’s structural growth appropriately while identifying significant changes with a Time Windows in Networks algorithm (Sulo et al., 2010). In each iteration, we divided the timeline into *T* windows of size *w*, and we created a graph time series *G_w_* = [*G*_1_, *G*_2_, …, *G_T_*], where *G_i_* represented the *Temporal network* subgraph that was observable in the *i^th^* window. Then, for each *G_i_* subgraph, we calculated the shortest path length statistic *f* (*G_i_*), due to its predominant role in calculating common centrality measurements (Wasserman & Faust, 1995). We constructed the statistical time series *F_w_* = [*f* (*G*_1_), *f* (*G*_2_), …, *f* (*G_T_*)], and we measured the noise caused by oversampling using the variance *V*(*F_w_*), and the redundant information across windows using the compress ratio *R* (*F_w_*). In order to calculate the compress ratio, we divided the length of *F_w_* by the length of $F_{w}^{*}$, which represents the statistical time series of the *Temporal network* subgraph composed by the set of nodes with more than 100 friends. We iterated the algorithm by varying the window size *w* from 1 month to the length in months of the timeline. Finally, we selected the optimal window size *w* that minimizes the absolute difference between *V*(*F_w_*) and *R*(*F_W_*). This procedure intended to divide the timeline into windows of equal size that could show relevant changes in the network, ensuring the minimum difference between the variance and the compress ratio of the statistical time series.

#### 2.5.2 Analyzing the human system growth of the program

Social networks are evolving dynamic human systems whose growth can occur based on different mechanisms (Newman, 2001). To understand the mechanism that leads to the emergence of cohesion and influential individuals, we evaluated if there was a preferential attachment in the Temporal network. Several studies indicate that popularity might be a key factor of friendship selection over time (Christakis & Fowler, 2007). In those cases, the growth mechanism is known as preferential attachment, or the “rich get richer” phenomenon, in which the new members of a social network tend to build friendships with people who have more ties, making them more connected over time (Barabási & Albert, 1999; Capocci et al., 2006).

We extracted the functional form of the preferential attachment function (Jeong et al., 2003), where each node with *k* ties will acquire new ties as an increasing function of *k* (Barabási & Albert, 1999). We defined *p_k_*(*t*) as the probability that a node with *k* ties in time *t* will establish a new friendship in time *t* + 1. Then, we estimated a relative probability *R_k_* by weighting *p_k_* (*t*) by a factor *N*(*t*)/*n_k_*(*t*) to avoid the bias created by the time dependence of the network growth.

(1) $$R_{k}=p_{k}(t)\cdot \frac{N(t)}{n_{k}(t)}$$

Here, *N*(*t*) is the amount of nodes in the network in time *t*, and *n_k_*(*t*) is the amount of nodes with *k* ties in time *t* (Newman, 2001). The slope of the function *R_k_* in relation to *k* will measure the attachment behavior. When the slope is less than one, there is a sublinear degree distribution as a stretched exponential; when the slope is equal to one, there is a degree distribution as a power law; and when the slope is greater than one, there is preferential attachment for one node (Jeong et al., 2003; Newman, 2001).

#### 2.5.3 Studying the scaling cohesion for the dynamical human system of the program

We determined if the growth of the dynamical human system was a proxy of cohesion among the Recreovía Facebook friends. We studied the network growth in terms of the relation among the number of nodes and edges per window. For this analysis, we adapted the scaling model of cities’ growth (Bettencourt et al., 2007):

(2) $$Y(t)=Y_{0}N(t{)}^{\beta }$$

where *Y*(*t*) represents the number of friendships at time *t*, and *N*(*t*) represents the number of stakeholders at time *t*. Then, we estimated *β* as a proxy measurement of cohesion. In the case of *β* < 1, we would consider a sublinear model with low cohesion, as the growth in the number of stakeholders is higher than the growth of ties among them. In the case of *β* = 1, we would consider a linear model with no change in cohesion, as the number of stakeholders and ties grow at the same rate. And in the case of *β* > 1, we would consider a super-linear model with high cohesion, as the growth in the number of stakeholders is lower than the growth of ties among them (Bettencourt et al., 2007).

## 3. Results

### 3.1 Assessing cohesion of the human system of the program with network measurements

**Figure 1.**
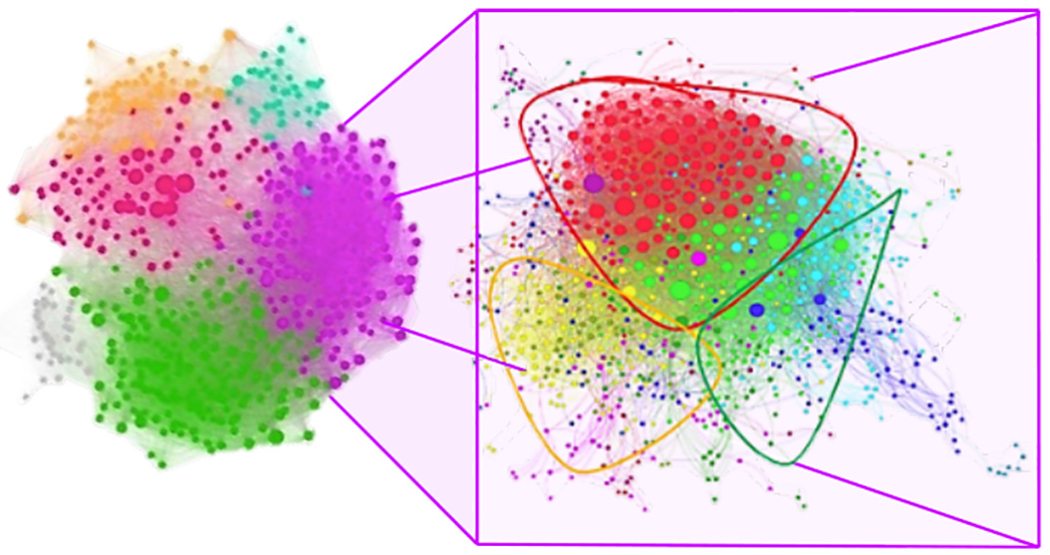
Left: The Main network. Each color represents a community detected with the maximum modularity algorithm of Louvain. The color scheme is as follows: green represents workers of fitness companies; gray represents the attendees of the program; orange represents city hall personnel; pink represents different institutions, organizations, and famous people; purple represents the physical activity instructors and other members of the Institute for Sports and Recreation (IDRD); and light blue represents other communities. Right: The physical activity instructors and other members of the IDRD community in the Main network. Each color represents a community detected with the maximum modularity algorithm of Louvain. The color scheme is as follows: red represents friends of the instructor WM; green represents friends of the instructor LH; and yellow represents friends of the instructor WP. This information was determined with IDRD members.

The first and static network, the Main network, was undirected and had 3,597 nodes and 51,361 edges, as shown in Figure 1. The sex of the nodes was available to be collected on Facebook in 2013. For the *Main network*, we observed 2,053 (57.06%) women, 1,423 (39.55%) men, and 121 (3.36%) non-reporting sex.

As a reflection of the social cohesion within the Recreovía, the *Main network* had a significantly higher clustering coefficient, higher diameter, and higher density compared with 100 random networks generated from the same size and degree distribution (Supplementary Material). The clustering of 0.41 indicates the probability that two friends of a specific node form a friendship among themselves. The diameter of 10 indicates the maximum number of contacts between each pair of nodes. And the density of 0.01 indicates a sparse network with a low ratio of existing edges over the possible ones. Those values characterize small-world networks, which have average low shortest paths among nodes, resulting in a cohesive network structure of stakeholders (Watts & Strogatz, 1998).

For the second and dynamic network, the Temporal network, we collected data in August 2016 for a timeline of 98 months (June 2008 to August 2016). The Temporal network was undirected, and it showed a cohesive longitudinal growth in size, clustering coefficient, diameter, and density over the timeline. These increments suggest that cohesion among stakeholders increased over time, but longitudinal paths also increased in length. The increments from the first month to the 98th were as follows: (1) 43–272 total nodes, (2) 55–2,565 total edges, (3) 0.19–0.21 clustering coefficient, (4) 4–7 diameter, and (5) 0.04–0.07 the density. For more details about changes in network metrics, please refer to Supplementary Material.

**Figure 2.**
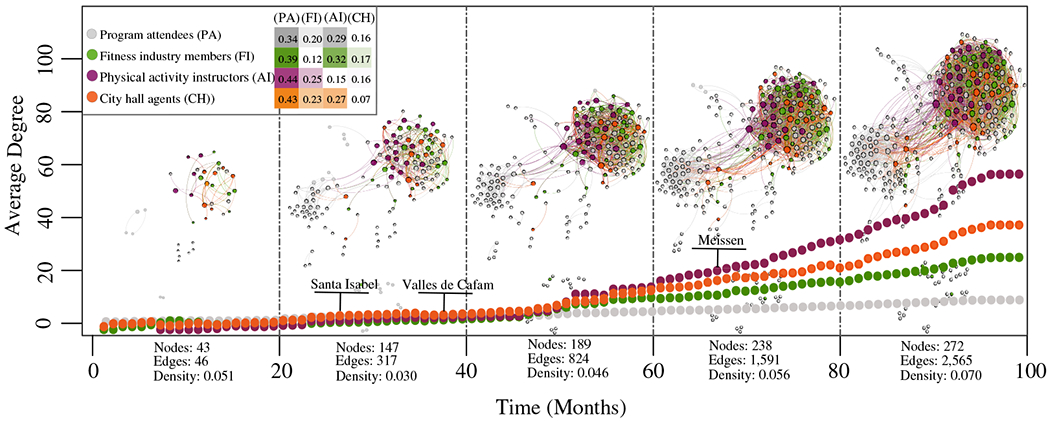
Growth of the *Temporal network* in windows of 20 months. The graphs represent the average degree of every category through the time, and the selected points with names are the first ties detected per each *Recreovía* station. The black dashed lines represent the time windows in which relevant growing events were detected. Each category was selected as the relation between the job descriptions in the Facebook profiles and the *Recreovía* program: The program attendees (PA) are those without relation; the fitness industry (FI) are those without relation with the program but related to fitness and health care centers; the physical activity instructors (AI) are those with a job description related to physical activity instructors of the program; and the city hall personnel (CH) are those with job description related to the administrative staff of the Institute for Sports and Recreation (IDRD). The colored table in the legend represents the percentage of ties among categories in relation to the number of ties per category (e.g., 0.34 is the number of relations PA-PA over the total PA relations); in this case, rows sum 1 and the darkest colors represent the most connected categories.

For the Temporal network, we categorized nodes according to the relationship between their job descriptions in the Facebook profiles and the Recreovía program. Categories were as follows: (1) program attendees, (2) fitness industry members, (3) physical activity instructors, and (4) city hall personnel (Figure 2).

Both networks presented cohesive patterns. The Main network had small-world properties compared with random networks of the same size and degree distribution, and the Temporal network had a growing cohesion reflected by the increment in size, connectivity, and probability of forming triads but with larger geodesic distances.

### 3.2 Identifying influential stakeholders and communities in the human system of the program

We detected the community structure of the Main network using the Louvain algorithm, obtaining 48 communities, of which 5 principal communities represented 83% of nodes in the Main network (left side of Figure 1). Principal communities’ names were as follows: (1) fitness companies, (2) attendees of the program, (3) city hall personnel, (4) different institutions, organizations, and famous people, (5) physical activity instructors, and (6) other communities. The largest community was the physical activity instructors, representing 20.84% of the network nodes. The sub-analysis made for this community suggested a greater cohesion compared with the *Main network* (right side of Figure 1). Accordingly, we identified that this community had a higher density (0.03) and clustering coefficient (0.53), and a smaller diameter (7) than the Main network (with values of 0.01, 0.41, and 10, respectively).

For clarity, from this point forward, we will refer to “*Name*” community for the communities detected by the Louvain algorithm in the Main network, and “Name” category for the categories identified by the job description in Facebook in the Temporal network.

As a sensitivity analysis, we characterized the influence of the physical activity instructors community in the *Main network* using the following centrality measures: degree, betweenness, closeness, k-core, and page rank. For the different measures, we found that nodes of the physical activity instructors community were always ranked as the most central nodes (Supplementary Material).

### 3.3 Assessing the emergence of social cohesion in the program over time

#### 3.3.1 Identifying time windows for the dynamical human system of the program

In the Temporal network subgraph (individuals with more than 100 friends), the most connected nodes were in the physical activity instructors category, representing 48.57% of nodes (Supplementary Material). These results are consistent with the Main network analyses.

We detected five time windows with the Time Windows in Network algorithm, each window with 20 months over the 98 months’ timeline. As a reflection of the cohesion within the Recreovía program, the connectivity of the nodes increased, being faster for the physical activity instructors and city hall personnel categories compared to the program attendees category. However, we found that the nodes in the program attendees category were the most important in the last window of the *Temporal network* as they were the most connected nodes in the ties of each category, more than 30%. The percentage of ties between each category and the attendees category was 34% in the program attendees category, 39% in the fitness industry members category, 44% in the physical activity instructors category, and 43% in the city hall personnel category (legend-table, Figure 2).

#### 3.3.2 Analyzing the human system growth of the program

**Figure 3.**
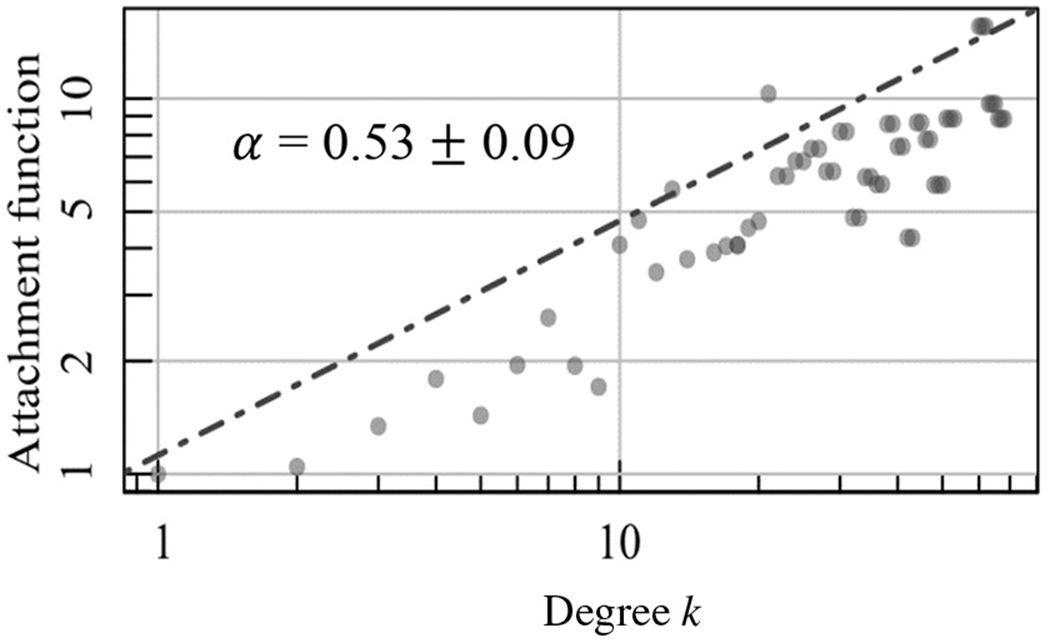
Preferential attachment function *R*_k_ in relation to K ties in the growth of the *Temporal network* through the time windows (Jeong et al., 2003; Newman, 2001). The *α* value is the slope of the graph.

We found that individuals were connected to popular and non-popular stakeholders without preference as a reflection of the cohesion growth among multiple stakeholders in the Recreovía network. Correspondingly, in the analysis of evolving networks, we found a sublinear preferential attachment behavior with a value of the slope *α* = 0.5± 0.09, which was lower than one (Figure 3), and the degree distribution among windows followed an exponential distribution (Newman, 2001).

For analyzing the results of the temporal methodology, we performed an additional analysis within each time window. This further analysis underscores the importance of the nodes in the physical activity instructors category inside each one of the last three windows, where the Temporal network shows a preferential attachment to the most popular nodes in the physical activity instructors category (Supplementary Material).

These results show the importance of studying the *Temporal network* as a dynamic process. The analysis of each window separately as a static network showed a particular preferential attachment to the most popular nodes in the physical activity instructors category in the last three windows. On the contrary, the analysis for evolving networks showed a non-preferential attachment behavior in the growth of the network through the time windows, revealing the emerging cohesion among all the stakeholders in the network—the last indicating that nodes were getting more connected without targeting some specific hubs.

#### 3.3.3 Studying the scaling cohesion for the dynamical human system of the program

**Figure 4.**
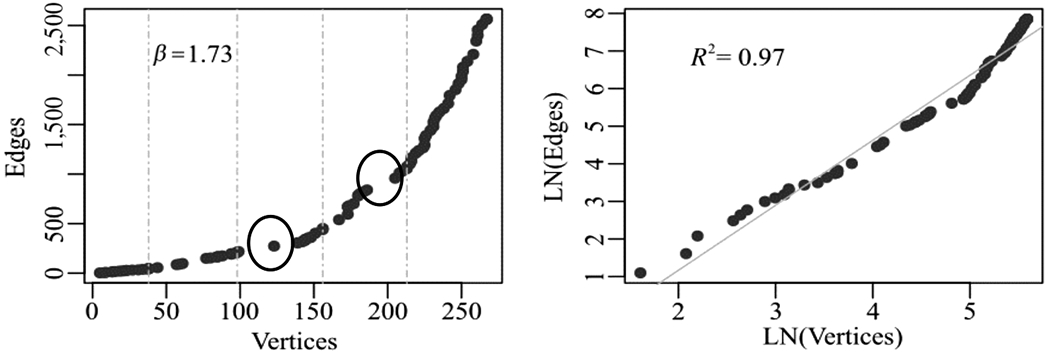
Temporal review of the aggregated social network of three Recreovía stations with the scaling model of city growth (Bettencourt et al., 2007). Left: Relation between number of friends and friendships in the Facebook profile of the program; dashed lines represent the time windows found with the Time Windows in Network algorithm. Two increase jumps in growth and highlighted in the graph. Right: Natural logarithm transformation of nodes and edges to correct scale problems.

The scaling model indicates an innovative growth of the Recreovía network. Specifically, when we applied the scaling model of cities to the Temporal network growth, we found a super-linear model (*β* = 1.73) that shows a higher growth of ties than the growth of the number of nodes (Figure 4). We detected two increases in the growth of ties compared to the growth of the number of nodes. We identified those increases as periods of innovation that are hypothesized to be related to (1) the implementation of new classes in the three Recreovía locations studied in this work (October, 2013), and (2) the marketing promotion strategies by IDRD through Facebook (November, 2014) (Supplementary Material).

#### 3.3.4 Surveys to the attendees of the program: physical and human system of *the program*

The survey respondents were 39 (78%) women and 11 (22%) men; 15 (30%) young adults in the (18–35 years) age range and 35 (70%) adults in the (≥35 years) age range; and 8 (16%) people in low socioeconomic income level, 28 (56%) people in low-middle socioeconomic income level, and 14 (28%) people in middle socioeconomic income level. The results of the surveys provided information about how the age range and location of attendees influenced the use of technological tools in the Recreovía program. Specifically, in Recreovía Santa Isabel, all interviewees were women older than 35 years (*n* = 6) and they had stronger preferences for using WhatsApp instead of Facebook as a communication tool. In Recreovía Valles de Cafam, women and men younger than 35 years (*n* = 15, 51%) were more supportive of using Facebook to gather information about the program because they used Facebook to interact with other attendees of the *Recreovía* and they were friends in Facebook of this *Recreovía* location.

## 4. Discussion

This study evaluated longitudinally the online social network of the Recreovía, a community-based program in Latin America that promotes healthy behaviors in public space. Our analysis underscores the social cohesion emerging as a co-benefit of the program, and the importance of physical activity instructors in the Recreovía. Both the structure of the Main network and the super-linear growth of the Temporal network were evidence of a cohesive structure of a system in which health and recreation are promoted. Also, we identified that the implementation of new classes in the parks and marketing promotion strategies through social media were associated with increases in the cohesion of the Temporal network. Future tracking of the Temporal network growth could anticipate sublinear regimes (when the number of stakeholders increase faster than their connectivity) and help to inform program managers conducting these types of interventions in relation to preserving the sustainable growth of the program.

We found that nodes in the physical activity instructors and IDRD stakeholders communities (for the *Main network*), and the physical activity instructors category (for the *Temporal network*) were the most connected nodes. In the Temporal network, most of the connections of the physical activity instructors were with the program attendees (≥0.30), as opposed to the number of edges representing instructor-instructor connections (0.15). The key role of the Recreovía’s physical activity instructors exemplifies the inviters’ and linkers’ roles identified in online social network studies (Kumar et al., 2006). These are individuals within the network that encourage offline friends and acquaintances to migrate online and to fully participate in the social evolution of the network (Viswanath et al., 2009). These findings underscore the importance of physical activity instructors as crucial actors to promote collaborative behaviors, and to be role models in the promotion and dissemination of the Recreovía program. These findings are consistent with a previous study in low- to moderate-income communities that found that health promoters have positive impacts on the attendance of a walking group program (Izumi et al., 2015). In that context of community-based interventions, social cohesion within groups was a mediator of the relationship between the leader behaviors and the exercise class adherence (Izumi et al., 2015).

Also, our results of the scaling growth of the Temporal network of the Recreovía program are consistent with scaling mechanisms observed in self-organized systems, including online social networks (Barabási & Albert, 1999) and community-based interventions. These scaling mechanisms are coherent with online social networks studies showing that the evolution characteristics of these types of networks present a persistent and well-connected core of nodes that become more interconnected in the evolving process fostering network cohesion (Kumar et al., 2006; Liu et al., 2014; Mislove et al., 2007). Regarding community-based interventions, other massive physical activity programs like Ciclovias have shown that regular participants reported higher social capital scores compared to irregular participants (Torres et al., 2013).

The limitations of our study include those related to the characteristics of online social networks, such as the limited knowledge of the core demographic characteristics from the Facebook profiles and the effects of unobserved data. Specifically, using Facebook as a data collection tool could be more appealing for individuals oriented towards online interactions, and we cannot argue direct causality of friendships in Facebook in response to the intervention. However, Facebook is a useful tool for studying social interactions within the Colombian and Latin American populations due to their high rates of using this social media. Currently, in Colombia, there are 31 million Facebook users (63% of an estimated 49 million population), with the largest audience within the 25–44 years range. Meanwhile, in South America as a whole, the overall penetration of social media is 83% (Digital in 2019, 2019). To better capture sociodemographic characteristics of the individuals within the network and diminish problems of unobserved data, future studies should implement longitudinal network surveys, radio-frequency identification, or mobile data.

The strengths of this study include: first, data collection from the entire online social network of a physical activity program and temporal data of three locations from low-income areas that have generally been under-studied; second, a longitudinal analysis of a program using both online social network data and personal interviews. Finally, it captures the scaling of an assessment of a public program in parks that allows for the identification of periods of innovation and a methodology for tracking its temporal network growth. This, in turn, can inform the program coordinators and sponsors about conducting interventions to preserve the sustainability of the program.

For future research, we suggest using growth models to estimate population and cohesion goals in online and offline networks of the program. This study could serve as a starting point for designing interventions aimed at promoting healthful behaviors in under-represented communities. These interventions might require a methodological approach that allows mapping and intervening in periodic offline social networks in the context of the physical activity programs. This methodological approach would allow for the measurement of further descriptive information about program attendees and the ties among them.

Our results could reinforce interventions of socially transmitted healthy habits among stakeholders and participants as they show the potential of online social networks to provide a greater understanding of the community dynamics of physical activity programs (Ferrer & Ellis, 2017; Rubio et al., 2020). This work serves as an example of an interdisciplinary systemic approach for promoting social and health dimensions of a community-based intervention occurring in parks in a low- to middle-income country.

## Supplementary Material

Supp material
