# Supplementary material for: Social cohesion emerging from a community-based physical activity program: A temporal network analysis
Source: Netw Sci (Camb Univ Press). Author manuscript; Available in PMC 2021 Jul 27. (PMC8315584; doi:10.1017/nws.2020.31)
Supplement: Supp material [file NIHMS1653458-supplement-Supp_material.pdf]

Supplementary material:

Table 1 Characteristics of the principal networks

Network name	Nodes	Edges	Density	Clustering coefficient	Average Path length
The main *Recreovía* Facebook Profile	3598	51361	0.008	0.407	3.04
The instructors of physical activity and members of IDRD community in the main *Recreovía* Facebook Profile	750	9864	0.035	0.53	2.66
The Aggregated Temporal *Recreovía* Facebook Profile	272	2565	0.07	0.212	2.26
The Compressed Aggregated *Recreovía* Facebook Profile	32	954	0.941	0.464	1.05

Statistical tests for the *Main network*:

We compared three topology metrics of the *Main network* with 100 random networks generated from the same size and same degree distribution using the configuration model generation.

1. Density:
   - Value in the *Main Network*: 0.008
   - Histogram of the random networks

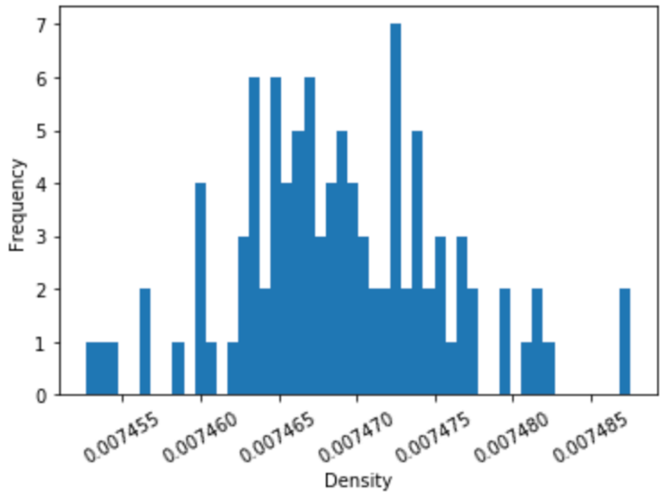

   - The statistical test of comparison of means $2.23 \times 10^{-184} < 0.05$ rejecting the null hypothesis of equal means, resulting in a higher density for the *Main network.*

2. Clustering coefficient:
   - Value in the *Main Network*: 0.407
   - Histogram of the random networks

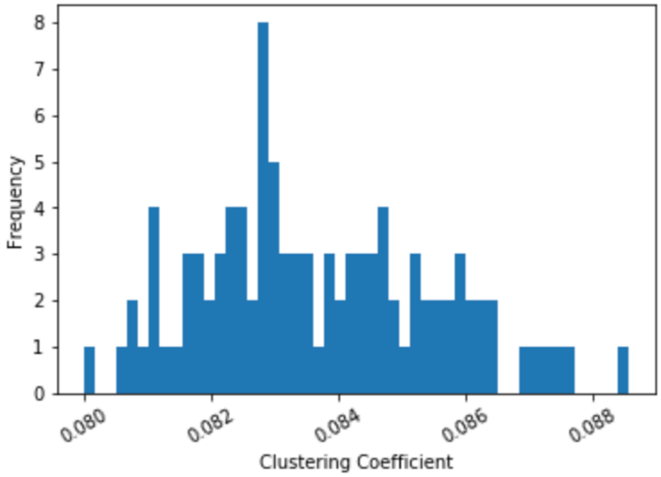

- The statistical test of comparison of means $2.23 \times 10^{-210} < 0.05$ rejecting the null hypothesis of equal means, resulting in a higher clustering for the *Main network.*

3. Diameter:
   - Value in the *Main Network*: 10
   - Histogram of the random networks

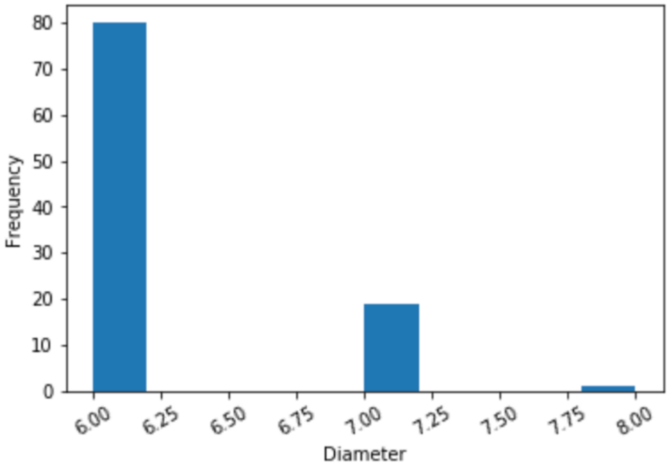

   - The statistical test of comparison of means $2.23 \times 10^{-95} < 0.05$ rejecting the null hypothesis of equal means, resulting in a higher diameter for the *Main network.*

Time windows in the temporal network

For the time TWIN algorithm, be created a compressed network in order to evaluate the redundant information in the *Aggregated Recreovía network.* We took those nodes with higher degree centrality, values greater than 100, to measure the ratio between the lengths of the time series statistic used in the procedure.

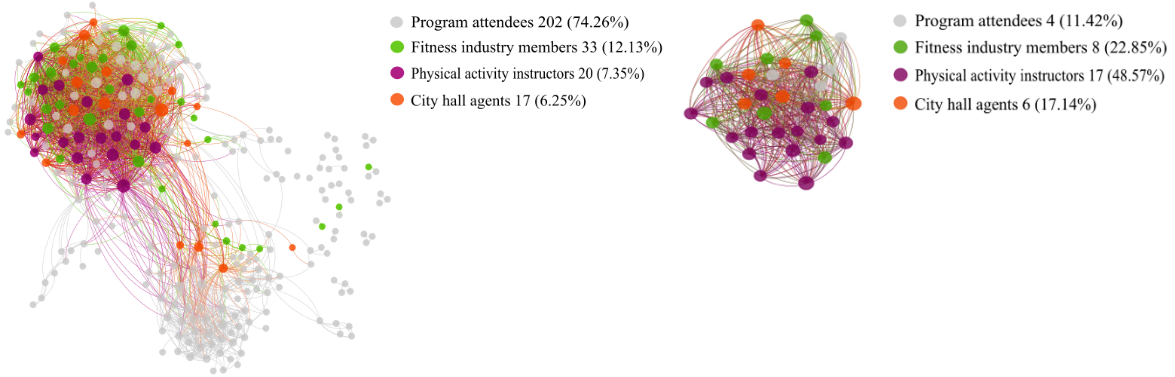

Figure 1 Left**:** The aggregated Facebook profile network of Recreovía Valles de Cafam, Recreovía Santa Isabel and Recreovía Meissen, where the size of nodes represents their degree. Right**:** The compressed aggregated Recreovía Facebook profile network, it is composed by those nodes with degree greater than 100.

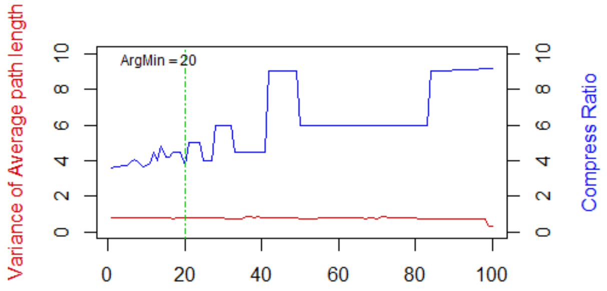

Figure 2 Results of all iterations of the Time Windows in Networks algorithm in the aggregated Facebook social network of three Recreovía program stations in Bogotá, where the minimum argument was found with windows size of 20 months, the green dashed line represents this minimum value.

Graph metrics for temporal networks

After finding the appropriate window size for the *Aggregated network,* we evaluated the growth of static centrality measures through the time windows in relation with topological behavior; and we implemented novel graph metrics for temporal networks(Holme, n.d.) in order to evaluate the time dependency of the network.

The centrality measures were: average degree, as the average number of friends that every node has; closeness coefficient, as the average shortest path length between each node and its friends; betweenness, as the frequency of appearance of each node in the shortest paths between all pair of nodes; diameter, as the maximum shortest path length in the network; density, as the number of real friendships over all possible friendships in the network; and the clustering coefficient, as the as the average probability that two friends of a node are themselves friends.(Introduction, n.d.) In addition, we calculated the degree distribution of the network through the time windows. That distribution is the probability $p(k)$ that each Facebook friend has k different friends(Introduction, n.d.), and we approximated different distributions to know the kind of theoretical network to which the *Aggregated network* is topologically equivalent.

The novel graph metrics were: the temporal global efficiency ε, as the quantify of robustness of the temporal graph.(Holme, n.d.)

$$\varepsilon = \frac{1}{N(N-1)} \sum_{ij} \frac{1}{d_{ij}}$$

Here N in the number of friends in the last window and d_{ij} is the temporal diameter between each pair of nodes i and j.

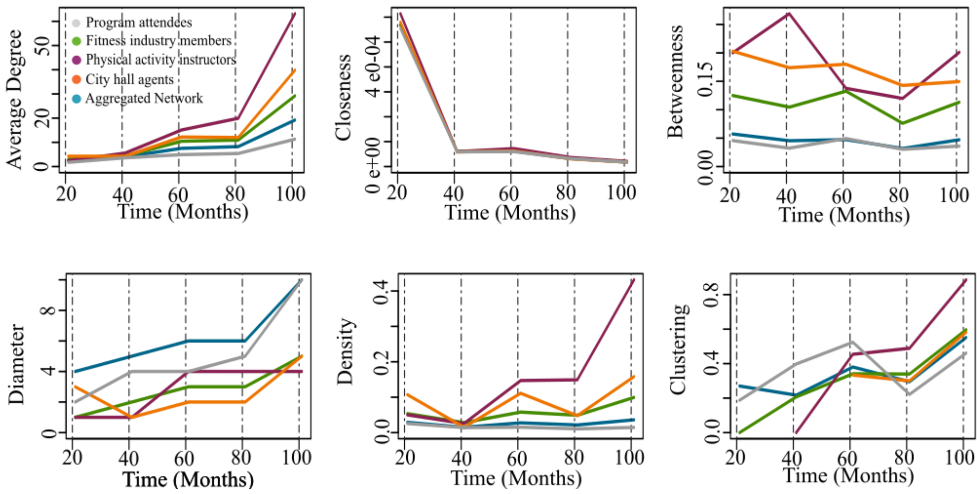

Figure 3 Evolution of centrality measures of the Aggregated network. Here each color represents the behavior of every community as an independent network and the blue color represents the entire network. The gray dashed lines represent the time windows where relevant growing events were detected.

We studied the topology of the ARFP network and we found that its degree follows a Power-Law distribution: $P(k) = C \cdot k^{-\lambda}$, (Introduction, n.d.) with a p-value of 0.99 in the Kolmogorov & Smirnov test to adjust distributions. (Figure 4)

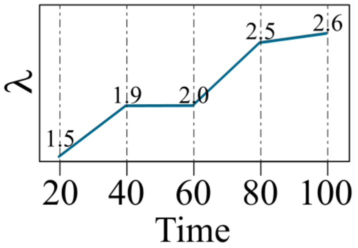

Figure 4 Power-Law degree distribution of the ARFP network where it follows a preferential attachment behavior where few nodes had the majority of connections and many nodes had connection amounts near to zero.

Table 2 Centrality measures of the principal influencers in the main Recreovía Facebook Profile

Influencers	Degree	Closeness centrality	Betweenness
WM	317	0.35	42741.26
WP	312	0.45	85636.68
LH	348	0.46	74149.57

Studying the scaling cohesion in time windows for the dynamical human system of the program

We evaluated the growth of the size and cohesion in the network during every time window (Figure 5) and we found two jumps in the growth of number of friends between consecutive months. Also we found a deaccelerated growth for network size in the last three windows ($\hat{\alpha}_3 = 2.7, \hat{\alpha}_4 = 1.5, \hat{\alpha}_5 = 1.7$) in relation with the slope in the second window ($\hat{\alpha}_2 = 4.4$) and we observed a linear slope for the transformed data with the natural logarithm. Similarly, we evaluated the growth of connections, and, in this case, it had an accelerated behavior in all time windows ($\hat{\alpha}_1 = 3.1, \hat{\alpha}_2 = 12.1,\ \hat{\alpha}_3 = 32.4, \hat{\alpha}_4 = 35.7, \hat{\alpha}_5 = 55.3$). We also observed a super linear slope in the number of connections transformed by the natural logarithm.

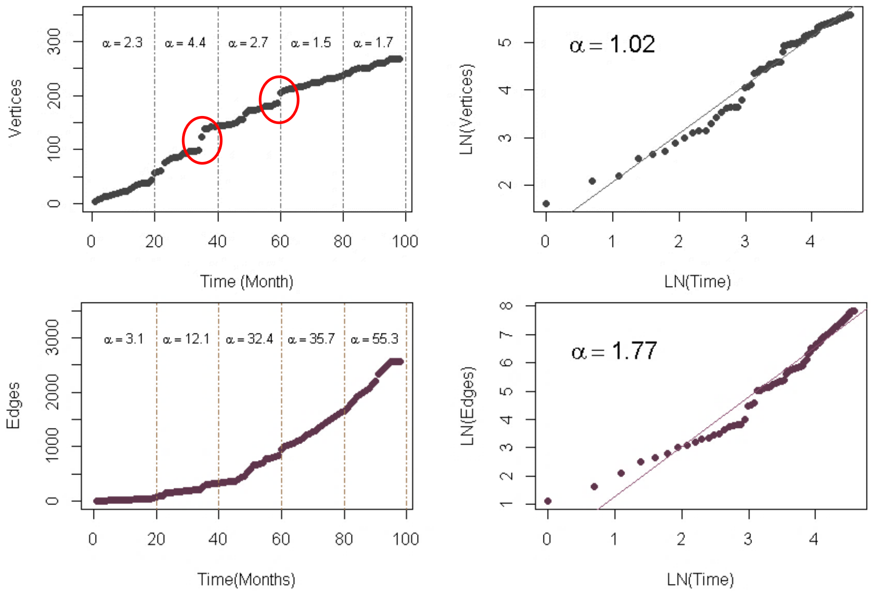

(WA)

Figure 4 Top left: Growth of the number of friends in the aggregated social network of the three Recreovía stations Facebook profiles above the 98 months, here dashed lines represent the time windows and the alpha values the slope in every window. Top right: Growth of the number of friends and time transformed with natural logarithm to correct the scale. Bottom left: Growth of the number of friendships in the aggregated social network above the 98 months, the dashed lines represent time windows and the alpha values the slop in every *window.* Bottom right: Growth of the amount of friendships and time transformed with natural algorithm to correct scale.

References

Holme, P. (n.d.). *Temporal Networks*.

Introduction, A. (n.d.). An Introduction.
